# Spanish Cross-Cultural Adaptation and Rasch Analysis of the Convergence Insufficiency Symptom Survey (CISS)

**DOI:** 10.1167/tvst.9.4.23

**Published:** 2020-03-23

**Authors:** Mariano González-Pérez, Carlos Pérez-Garmendia, Ana Rosa Barrio, María García-Montero, Beatriz Antona

**Affiliations:** 1 Optics and Optometry Department, Faculty of Optics and Optometry, Complutense University of Madrid, Madrid, Spain; 2 Applied Vision Research Group, Faculty of Optics and Optometry, Complutense University of Madrid, Madrid, Spain

**Keywords:** convergence insufficiency, patient-reported outcomes, CISS, Rasch analysis, cross-cultural validation

## Abstract

**Purpose:**

To culturally and linguistically adapt the Convergence Insufficiency Symptom Survey (CISS) to Spanish and assess the psychometric performance of the new version through Rasch analysis and classical test theory methods.

**Methods:**

The Spanish version of the CISS (CISS_VE_) was completed by 449 subjects (9–30 years old) from the general population. The validity and reliability of CISS_VE_ were assessed through Rasch statistics (precision, targeting, item fit, unidimensionality, and differential item functioning). To test construct validity, we calculated the coefficients of correlation between the CISS_VE_ and the Computer-Vision Symptom Scale (CVSS17) or Warwick–Edinburgh Mental Well-Being Scale (WEMWBS). We determined test–retest reliability in a subset of 229 subjects. We used differential item functioning (DIF) to compare the CISSS_VE_ and the CISS after administering the CISS to 216 English children.

**Results:**

After applying exclusion criteria, the responses of 420 participants (mean age, 18.62 years; female, 54.95%) revealed good Rasch model fit, good precision (person separation = 2.33), and suboptimal targeting (–1.37). There was some evidence of multidimensionality, but disattenuated correlations between the Rasch dimension and a possible secondary dimension were high, suggesting they were measuring similar constructs. No item bias according to gender or age was detected. Spearman's correlation was 0.34 (*P* < 0.001) for CISS_VE_–CVSS17 and non-significant for CISS_VE_–WEMWBS. The limits of agreement for test–retest reliability were 9.67 and –8.71. Rasch analysis results indicated no difference between CISS and CISS_VE_.

**Conclusions:**

According to our results, CISS_VE_ is a valid and reliable tool for measuring the symptoms assessed by CISS in Spanish people 9 to 30 years of age.

**Translational Relevance:**

CISS_VE_ can measure convergence insufficiency symptoms in Spanish-speaking subjects.

## Introduction

Convergence insufficiency is one of the most common abnormalities of binocular vision. It is usually associated with symptoms such as visual fatigue, headaches, blur, and double vision.^1^^,^^2^ To measure these symptoms, the Convergence Insufficiency Symptom Survey (CISS) was developed in 1999 by the Convergence Insufficiency and Reading Study Group (CIRS).^3^

The first version of this questionnaire consisted of 13 items and assessed the frequency of each symptom using a four-option response scale.^3^ In 2003, a revised version was introduced^1^ that included two more items and a new response scale with five choices: never, infrequently, sometimes, fairly often, and always. This new version made the tracking of changes during therapeutic interventions more sensitive.^1^ The 15-item version of the CISS (hereafter CISS) is a frequently used outcome measure in binocular vision research and has been used to assess convergence insufficiency (CI) symptoms in various clinical groups from the ages of 8 to 30 years,^1^^,^^2^^,^^4^ where subjects with symptomatic CI had a significantly higher CISS score than others with normal binocular vision. However, to our knowledge, no data have been reported regarding its psychometric properties apart from its repeatability^2^ and known-group validity.^3^^,^^4^ This last variable reflects the ability of a questionnaire to discriminate between two groups known to differ a priori.

The CISS is not a condition-specific instrument for convergence insufficiency; rather, it is useful for measuring the symptoms associated with visual discomfort caused by different factors. Accordingly, it considers the most common symptoms regarding near-vision problems^5^ and provides similar scores in children with accommodative insufficiency and convergence insufficiency.^4^ In addition, as described by Horan et al.,^6^ some patients with normal sensorimotor exam results were found to score high (i.e., showed a higher level of the assessed symptoms) on the CISS, while others with convergence insufficiency had relatively low scores.

The CISS was developed for English speakers. As there are 442 million native Spanish speakers worldwide, there is currently a need for a Spanish version. We generated a Spanish version of the CISS (CISS_VE_) following well-known guidelines^7^^–^^11^ used for other recent cross-cultural adaptations^12^^,^^13^ to ensure content and operational equivalence between the original CISS and the CISS_VE_. Most cross-cultural adaptation studies are based on modern psychometric models such as the Rasch item response theory (IRT) model. This model is recommended for the quality assessment of health questionnaires because (1) it generates a more precise measure, overcoming the limitations of traditional summary scoring through the transformation of ordinal raw scores into interval linear scales^14^^–^^17^; and (2) it provides insight into the psychometric properties of the scale and is able to match item difficulty to user skills.^15^ The Rasch approach also provides data, such as person and item reliability, reflecting the overall performance of the instrument.^16^ The objective of this study was to culturally and linguistically translate the Spanish version and assess its psychometric performance using Rasch analysis and classical test theory methods.

## Methods

Before the study outset, the authors of the CISS gave us their consent to develop a Spanish version of their instrument. The CISS questionnaire consists of 15 items. In reply to each question, the subject indicates the frequency of each symptom on a Likert scale, with scores ranging from 0 to 4: never (0), infrequently (1), sometimes (2), often (3), or always (4). The scores of every item are added to determine the final score, which ranges from 0 (least symptomatic) to 60 (most symptomatic). The recommended cut-off is ≥21 for adults^2^ and ≥16 for children 9 to 18 years of age.^1^ The study was conducted in two stages. The questionnaire was first translated and adapted to Spanish (May 2016 to April 2017), and then the validity and repeatability of the Spanish version were assessed (May 2017 to January 2018).

Because the CISS assesses near-vision-related symptoms and not only convergence insufficiency symptoms, we enrolled subjects from the general population from four different institutions in Madrid, Spain: a primary school (CEIP Vargas Llosa, Madrid), a secondary school (IES Juan Rodriguez Villanueva), a university faculty (Optics and Optometry Faculty of the Universidad Complutense de Madrid), and a technology company (DXC). In addition, over the period from January 2019 to March 2019, we performed a psychometric analysis of the English CISS on subjects from the personal network of one of our researchers (CP-G) in Swindon, Wiltshire, UK. Subjects 9 to 30 years of age were recruited by convenience. Participants received no compensation for their cooperation. Exclusion criteria were mother tongue different from the questionnaire language, prior visual surgery (not refractive), active visual or neurologic disease, any medication that could affect vision, or any kind of disability preventing the subject from reading or understanding the instrument's questions. Out of 665 subjects enrolled, 449 (mean age, 18.62 years; range, 9–30 years; female, 54.95%) completed the Spanish version of the CISS (CISS_VE_), and 216 (mean age, 15.81 years; range, 12–20 years; female, 37.61%) completed the original CISS.

The study was approved by the Research Ethics Committee of the Hospital Clínico San Carlos (Madrid, Spain), and its protocol adhered to the tenets of the Declaration of Helsinki. All participants gave their written informed consent prior to participation. For participants younger than 18 years, consent was obtained from a parent or guardian. All children older than 12 years also provided their consent before any testing was done. Other than responses to the questionnaires, no other clinical data were collected in any subject.

### Translation and Transcultural Adaptation of CISS

Translation and adaptation were performed according to previously published guidelines^7^^,^^18^^,^^19^ as a five-step process:

1. *Direct translation*—Two bilingual translators with Spanish as their mother tongue, one member of the research group (CP) and another person working in a different area of knowledge (banking), independently translated the original CISS version. CP provided clinical equivalence with the original CISS, and the other translator offered a vernacular version.
2. *Consensus version of the direct translation*—The two bilingual translators ensured that the translation was fully comprehensible.
3. *Back translation*—A further two bilingual translators, this time with the source language (English) as their mother tongue and who were blind to the original version, independently translated the consensus version back into Spanish. Both translators were naive about the concepts explored to avoid information bias.^7^
4. *Expert committee review*—The expert panel included all of the translators, one who was a professional translator involved in the research, two who were experts in binocular vision (AB and BA), and a team member who had a background in patient-reported outcome instrument development (MG-P). The panel held a meeting to consolidate the four previous translations and created the pre-final version of the Spanish CISS, designated CISS-*versión* *española*, or CISS_VE_. The panel solved discrepancies by consensus, and a written summary of this meeting is provided as Supplementary Table S1. As an example, after discussing whether the response option “fairly often” should be translated as “casi siempre” or “bastante a menudo,” the latter translation was finally adopted by consensus.
5. *Pre-testing of the consensus version*—Cognitive interviews were conducted using a verbal probing technique with 48 native speakers between the ages of 9 and 30 years to ensure patient comprehension of the CISS_VE_; no new issues emerged in this pre-test.

### Analysis Strategy

For descriptive data generation and repeatability assessment, we used SPSS Statistics 22.0 (IBM Corp., Armonk, NY, USA).

### Rasch Analysis

The package Winsteps 4.0.1 (Winsteps.com, Beaverton, OR, USA)^7^ was used for Rasch analysis. The Rasch model is an IRT that transforms raw scores to express the person ability and the difficulty of items on the same scale, so that the difference between the ability of two people does not depend on the specific items with which their ability is estimated. The main IRT concept is that a mathematical model is used to predict the probability of a person successfully replying to an item according to person ability and item difficulty.^20^ For the analysis, we chose the Andrich rating scale model (RSM), which assumes equal category thresholds across items, as all items share the same response option structure.^21^ Respondents with a greater level of symptoms and items of greater difficulty were located on the negative side of the continuum scale and vice versa. The results of the Rasch method were then used to determine the following.

#### Rating Scale Structure

The performance of the rating scale structure was assessed by examining the category threshold order. Disordering of categories occurs when the response options do not follow expected hierarchical ordering.^13^^,^^22^

#### Item Fit Statistics

Both infit and outfit mean square fit statistics show the extent to which the items in the domain comply with Rasch model expectations.^14^

#### Dimensionality

The scale is considered unidimensional when there is one latent variable of interest, and the level of this latent variable is the focus of measurement.^20^ To assess multidimensionality, we used the results of the Rasch principal component analysis (PCA) of standardized residuals, which looks for patterns in the part of the data that does not agree with the Rasch measures (unexpected data). When groups of items share the same patterns of unexpected data, those items probably also share a substantive attribute in common, which we refer to as a “secondary dimension” or “contrast.”^23^ When variance explained by the Rasch measures is ≤50% and/or the eigenvalue in the first contrast is ≥2.0, this is an indication of subsets of items that suggest multidimensionality.^14^ Next, we looked at the disattenuated coefficient of correlation between the first and second contrasts obtained in the PCA analysis. Disattenuated correlation approximates the correlation between the two contrasts without measurement error. According to Linacre,^24^ 0.82 may be used as the cut-off to consider that the two contrasts measure the same variable.

#### Person Separation Index and Levels of Performance

The Rasch-based Person Separation Index (PSI) is a reliability indicator, analogous to Cronbach's α of traditional test theory in both its values and construction.^25^ This index was obtained using Winsteps. The number of different levels of performance was computed according to the method described by Wright.^21^^,^^26^

#### Targeting

The extent to which item difficulty, defined as the point on the latent variable at which the highest and lowest category of an item have equal probability of being observed, matches the level of a participant's visual abilities was defined as the difference between the average difficulty of the items and the subject's mean level of symptoms.^14^

#### Differential Item Functioning by Gender, Age Group, and CISS Version

We examined each item to determine if there was any difference in the way subgroups (male vs. female; children under 18 years of age vs. young adults older than 18) answered each item—that is, no differential item functioning (DIF). In addition, because testing for DIF is a useful way to validate questionnaire translations,^27^ we performed this analysis to test whether the CISS_VE_ items were equivalent to those of the original survey. Accordingly, the DIF for an item was considered a cross-cultural or translational issue for that particular translation.^28^

The DIF analysis implemented in Winsteps is based on two methods:

1. Mantel–Haenszel method to estimate the log odds of DIF size and significance from cross-tabs of observations in the two groups
2. Logit-difference (logistic regression) method to estimate the difference between Rasch item difficulties for the two groups, maintaining everything else constant^24^

DIF contrast (i.e., difference in difficulty of the item between the two groups) was defined as no-DIF for <0.50 logits, minimal for 0.50 to 1.0 logits, and notable for >1.0 logits.^13^

The overall quality of the psychometric data obtained in this stage (except levels of performance) was assessed according to the criteria of the guidelines proposed by Khadka et al.^14^ for quality assessment of ophthalmologic questionnaires.

### Validity and Repeatability

Paper versions of the CISS_VE_, the Computer-Vision Symptom Scale (CVSS17),^26^ and the Warwick–Edinburgh Mental Well-Being Scale (WEMWBS)^29^ were administered to all participants except the primary-school children. Seven days later or longer, subjects again completed the CISS_VE_ in a second session. Convergent validity was assessed by estimating the coefficient of correlation between the subjects’ CISS_VE_ and CVSS scores and divergent validity through the coefficient of correlation between CISS_VE_ and WEMWBS scores. According to Khadka et al.,^14^ a coefficient of correlation between CISS_VE_ and CVSS greater than 0.3 would be considered as proof of convergent validity. The Kolmogorov–Smirnov test used on the CISS_VE_, CVSS, and WEMWBS scores indicated a non-normal distribution of all measures, so we calculated the Spearman's rho coefficient of correlation. The repeatability of the CISS_VE_ was examined via the intraclass correlation coefficient (ICC) with the confidence interval set at 95%. In addition, Bland–Altman limits of agreement were determined to calculate the coefficient of repeatability (CoR) by subtracting the mean difference in scores between the two CISS_VE_ sessions from the upper 95% limit.^30^

## Results

### Spanish Version of the CISS

Table 1 shows the CISS_VE_ items and response descriptors emerging from the pre-test administered to 48 subjects (33.33% female) and the corresponding items and descriptors taken from the original CISS.

**Table 1.** Items and Response Options for the Original Convergence Insufficiency Symptom Survey and the Spanish Version

	Convergence Insufficiency Symptom Survey (CISS)	Escala Sobre Síntomas de Insuficiencia de Convergencia (CISS_VE_)
	Original Version (US English) Items	Translated (European Spanish) Items
Instructions	Please answer the following questions about how your eyes feel when reading or doing close work.	Las siguientes preguntas se refieren a cómo se siente mientras lee o trabaja de cerca.
Questions		
1	Do your eyes feel tired when reading or doing close work?	¿Notas tus ojos cansados?
2	Do your eyes feel uncomfortable when reading or doing close work?	¿Notas incomodidad en tus ojos?
3	Do you have headaches when reading or doing close work?	¿Te duele la cabeza?
4	Do you feel sleepy when reading or doing close work?	¿Te entra sueño?
5	Do you lose concentration when reading or doing close work?	¿Pierdes la concentración?
6	Do you have trouble remembering what you have read?	¿Te cuesta recordar lo que has leído?
7	Do you have double vision when reading or doing close work?	¿Ves doble?
8	Do you see the words move, jump, swim, or appear to float on the page when reading or doing close work?	¿Te parece que las palabras se mueven, se mezclan o flotan sobre el texto?
9	Do you feel like you read slowly?	¿Te parece que lees lento?
10	Do your eyes ever hurt when reading or doing close work?	¿Te duelen los ojos?
11	Do your eyes ever feel sore when reading or doing close work?	¿Se te irritan los ojos?
12	Do you feel a “pulling” feeling around your eyes when reading or doing close work?	¿Tienes sensación de “tirantez” alrededor de los ojos?
13	Do you notice the words blurring or coming in and out of focus when reading or doing close work?	¿Notas que las palabras se ponen borrosas o que se enfocan y desenfocan?
14	Do you lose your place while reading or doing close work?	¿Te pierdes de línea al leer?
15	Do you have to re-read the same line of words when reading?	¿Tienes que releer la misma línea de texto?
Translation of response categories	0, never; 1, infrequently; 2, sometimes; 3, fairly often; 4, always	0, nunca; 1, muy pocas veces; 2, algunas veces; 3, muchas veces; 4, siempre

### Rasch Analysis

Of the questionnaires completed by 449 participants, responses to 429 questionnaires (mean age, 15.92 ± 5.59 years; 55.2% female) were used in the Andrich's rating scale model (RSM) analysis implemented in Winsteps. The reasons for excluding 20 of the completed questionnaires from analysis were that more than 33% of the items were not answered by one participant, and outfit > 2.5^26^^,^^31^ in the responses provided by 19 participants (outfit is sensitive to unexpected observations by persons on items^24^). The mean CISS_VE_ score obtained was 15.10 ± 10.13, and the range was 1 to 50.

#### Rating Scale Structure

**Figure 1.**
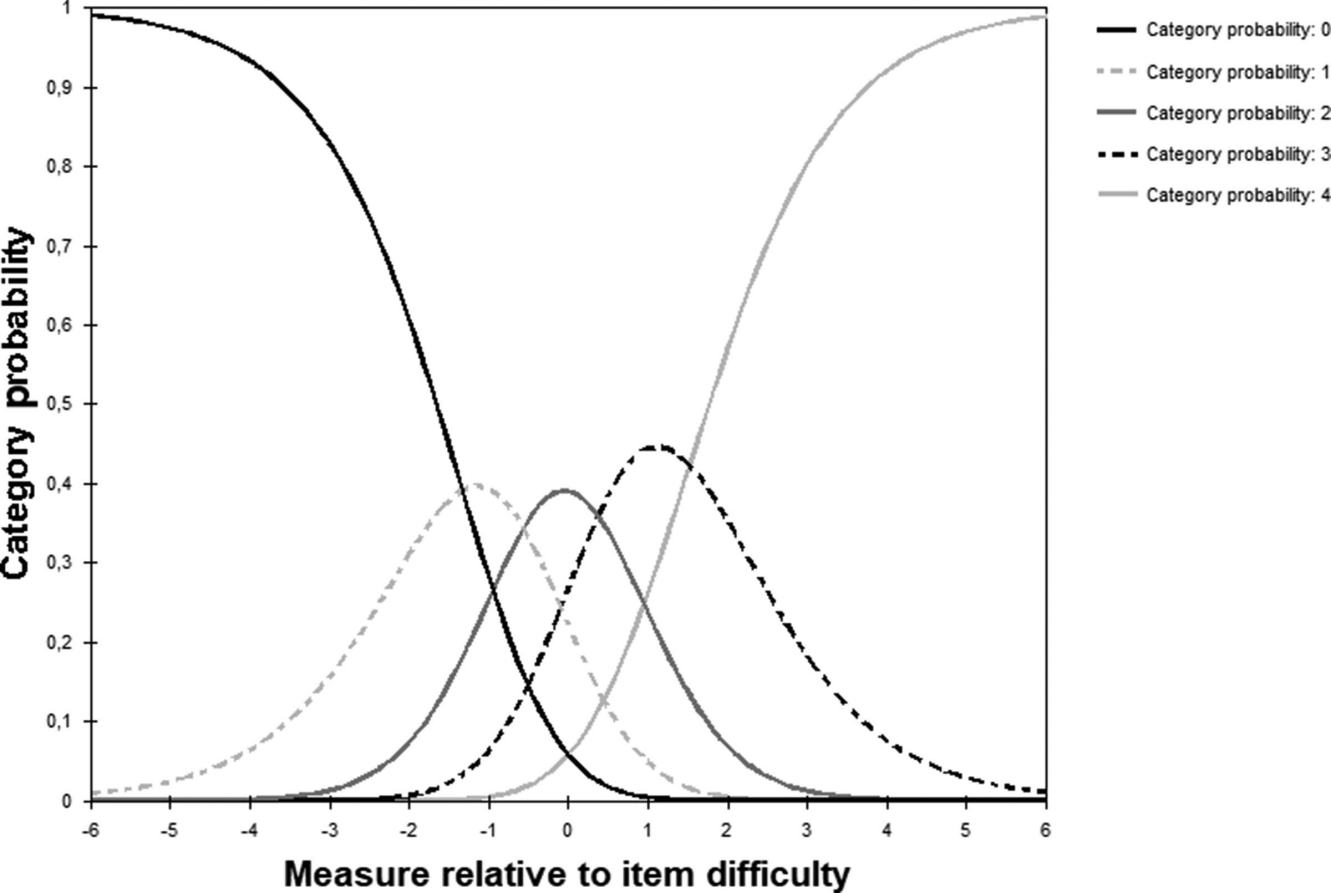
Category probability curves of the CISS_VE_. The figure shows the performance of the five response categories of the CISS_VE_, which asked about the frequency for each of the assessed symptoms. The curve at the extreme left represents “never,” and the curve at the extreme right represents “always.”

There was no disordering of response categories (Fig. 1).

#### Item Fit Statistics

Item fit statistics and item measure (difficulty, in logits) for the CISS_VE_ are provided in Table 2. All items showed values inside the interval considered productive for measurement.^13^^,^^17^ Only the infit and outfit of item 1 were outside the more stringent criterion (0.7–1.3) proposed by Pesudovs et al.^16^ and Khadka et al.^14^

**Table 2.** Rasch Fit Statistics and Item Measure for CISS_VE_

		Infit	Outfit	Measure
Item No.	Item Descriptor (in US English)	(MNSQ)	(MNSQ)	(Logits)
1	Do your eyes feel tired when reading or doing close work?	0.59	0.6	−0.68
2	Do your eyes feel uncomfortable when reading or doing close work?	0.76	0.74	−0.19
3	Do you have headaches when reading or doing close work?	1.2	1.26	−0.55
4	Do you feel sleepy when reading or doing close work?	1.19	1.14	−0.8
5	Do you lose concentration when reading or doing close work?	1.08	1.07	−0.53
6	Do you have trouble remembering what you have read?	1.18	1.15	−0.07
7	Do you have double vision when reading or doing close work?	1.1	1.01	1.17
8	Do you see the words move, jump, swim, or appear to float on the page when reading or doing close work?	1.13	0.9	0.95
9	Do you feel like you read slowly?	1.13	1.13	0.47
10	Do your eyes ever hurt when reading or doing close work?	0.79	0.77	−0.15
11	Do your eyes ever feel sore when reading or doing close work?	1.03	0.96	0.03
12	Do you feel a “pulling” feeling around your eyes when reading or doing close work?	1.17	1.12	0.23
13	Do you notice the words blurring or coming in and out of focus when reading or doing close work?	0.93	0.96	0.07
14	Do you lose your place while reading or doing close work?	1.09	1.09	−0.05
15	Do you have to re-read the same line of words when reading?	0.95	0.94	0.09

MNSQ, infit/outfit mean square.

#### Dimensionality

Our PCA analysis of the CISS_VE_ revealed that 46.3% of the raw variance was explained by the CISS_VE_ measures, and an eigenvalue of the first contrast of 2.19. All other contrasts had eigenvalues below 2.00. Thus, in our analysis, the secondary dimension was noticeable because it was bigger than 2.0, indicating that the CISS_VE_ measures two different latent traits. Table 3 shows the items covering the secondary dimension. The disattenuated coefficient of correlation between the first and second contrasts was 0.84, indicating that both dimensions share about 67% of the person measure variance. Table 4 summarizes the results of the tests used to assess unidimensionality and how we used these data to decide whether the CISS_VE_ could be considered unidimensional. According to these results, the CISS_VE_ can be considered a unidimensional instrument.

**Table 3.** Items Comprising the Second Dimension

		Standardized
		Residual
	Item Descriptor	Loading
5	Do you lose concentration when reading or doing close work?	0.53
6	Do you have trouble remembering what you have read?	0.47
15	Do you have to re-read the same line of words when reading?	0.45
14	Do you lose your place while reading or doing close work?	0.42
9	Do you feel like you read slowly?	0.35
4	Do you feel sleepy when reading or doing close work?	0.28

Item descriptors (in English) are ordered by first contrast loading as defined by Rasch-residual-based PCA analysis.

**Table 4.** Summary of the Multidimensionality Test Results and Their Interpretation

Multidimesionality		
Tests	Result	Interpretation
PCA analysis: percent of raw variance explained by the measure	46.3	Suggests multidimensionality
PCA analysis: eigenvalue of the unexplained variance in the first contrast	2.19	Suggests multidimensionality; secondary dimension was noticeable
Disattenuated coefficient of correlation between the first and second contrasts obtained from PCA analysis	0.84	Rules out dimensionality

#### Person Separation Index and Performance Levels

**Figure 2.**
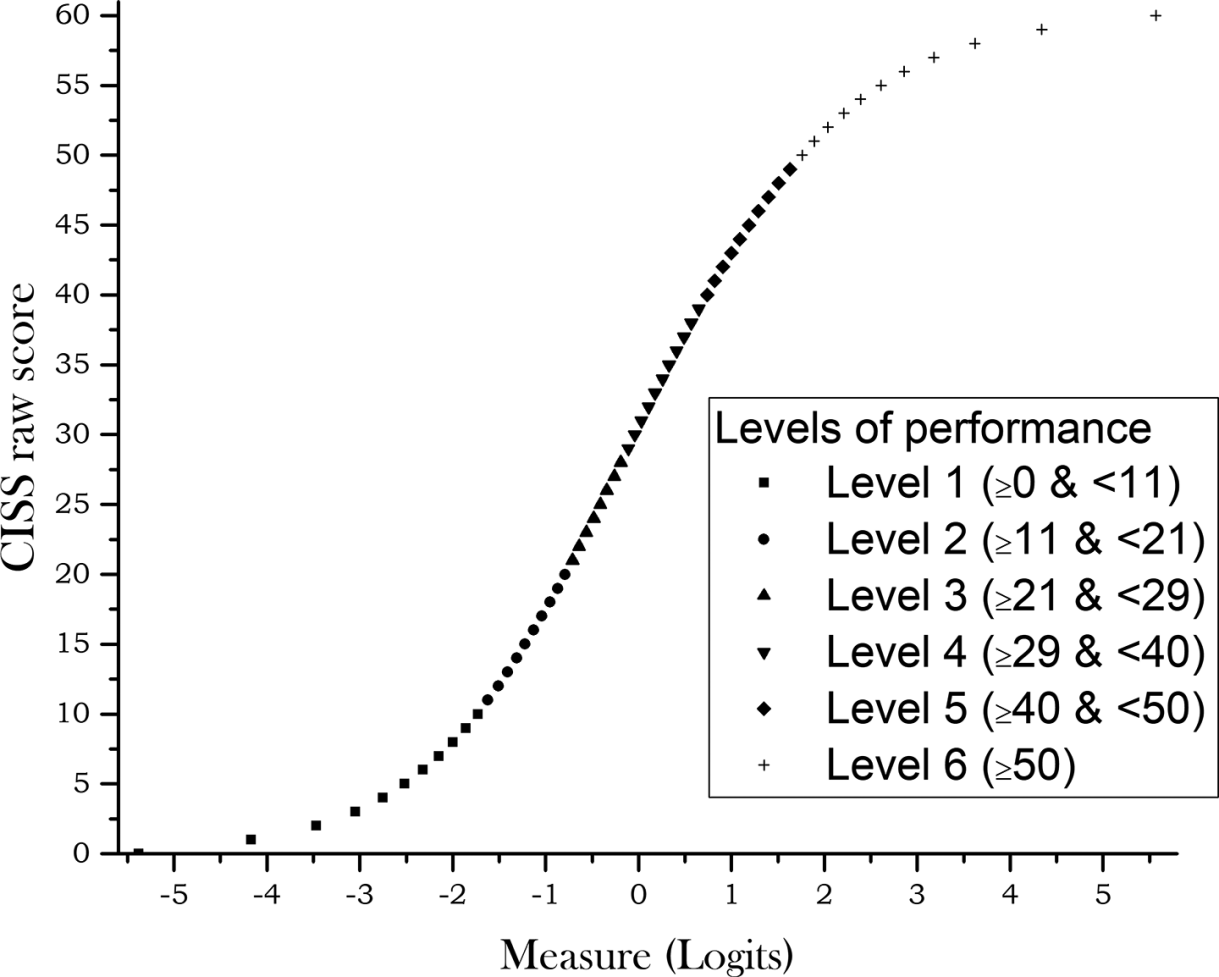
Plot of estimated measure (*x*-axis) for any CISS_VE_ raw score (*y*-axis). Different symbols represent distinct levels of performance as indicated in the figure inset.

The PSI for CISS_VE_ was 2.33, indicating a reliability of 0.85 and meaning that the CISS_VE_ was able to distinguish 3.44 strata of scores. Using the Wright method (a sample-independent method suitable for clinical samples) to determine the number of performance levels across the CISS_VE_ score range, we found that the CISS_VE_ could distinguish 6.3 levels of symptoms. Figure 2 shows the estimated measure for any CISS_VE_ raw score and the correspondence between the raw score and level of performance. Cronbach's α was 0.90. Table 5 shows the distribution of the CISS_VE_ and CISS scores obtained by the subjects included in this study according to performance level.

**Table 5.** Demographic and Score Distributions for the 637 Sets of Responses Analyzed in Our Study

	Total (N = 637)	CISS_VE_ (n = 429)	CISS (n = 208)
Mean age ± SD	15.90 ± 4.71	15.92 ± 5.59	15.86 ± 1.61
Female proportion	49.8%	55.2%	38%
Score range	1–50	1–50	1–49
Score, mean ± SD	15.86 ± 9.74	15.10 ± 10.13	16.10 ± 9.50
Score median, IQR	15.8–29	14.8–30	16.1–28.1
Level of symptoms, %			
Level 1	33.12	35.20	28.85
Level 2	38.93	38.23	40.38
Level 3	17.11	15.15	21.15
Level 4	8.95	9.32	8.17
Level 5	1.73	1.86	1.44
Level 6	0.16	0.23	0

SD, standard deviation; IQR, interquartile range.

#### Targeting

**Figure 3.**
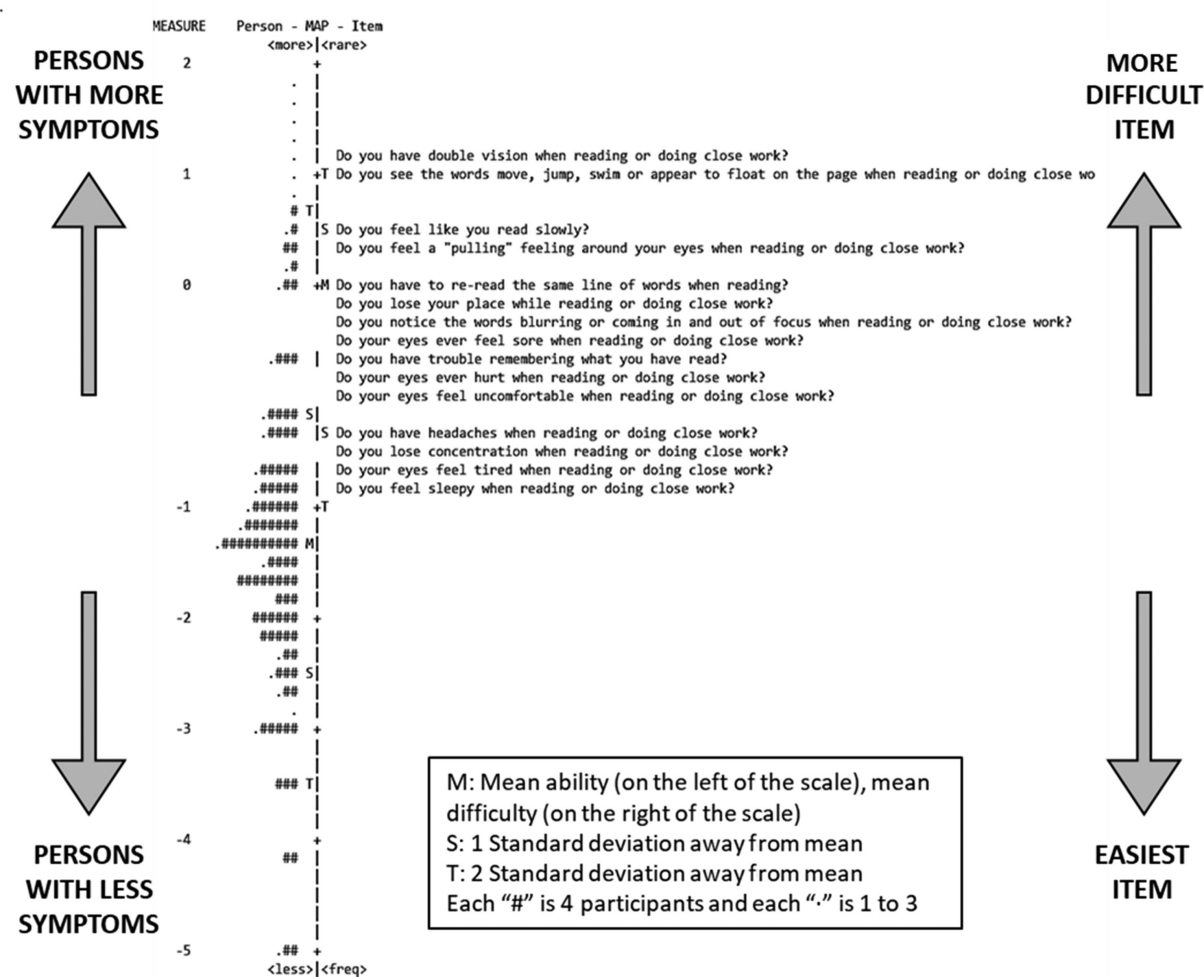
Item-person map for the CISS_VE_. The Rasch item-person map orders the self-reported level of symptoms of the patients in our study (left side) and the item difficulty (right side).

The targeting value was –1.37 logits. The item-person map (Figure 3) shows that the items were too difficult for the ability level in this sample, because we assessed a population-based sample in which most were not expected to have near-vision symptoms.

#### Differential Item Functioning by Gender and Age

The results of DIF by gender revealed neither notable DIF nor minimal DIF for any of the CISS_VE_ items. Just one item (item 14) showed minimal DIF (0.78) according to age group, as this item was more difficult for young adults than for children. To assess the psychometric properties of CISS_VE_, we compared our Rasch analysis results against the Rasch model expectation^22^ using the quality criteria proposed by Khadka et al.^14^ (Table 6).

**Table 6.** Psychometric Properties of the CISS_VE_

Parameter	Rasch Model Expectation	CISS_VE_
Number of items	–	15
Response categories ordering	Ordered	Ordered
Person separation index (reliability)	>2.0 (>0.80)	2.33 (0.85)
PCA		
Raw variance explained by measure, %	>50	45.8
Eigenvalue of the first contrast, logits	<2.0	2.18
Number of items with infit outside 0.7 to 1.3	0	1
Number of items with outfit outside 0.7 to 1.3	0	1
Number of items with DIF for gender of <0.5 logits and *P* > 0.05	None	None
Number of items with DIF for age group of <0.5 logits and *P* > 0.05	None	1
Targeting	≥−1.0	−1.37

PCA, principal component analysis; DIF, differential item functioning.

#### English Version Versus Spanish Version

For the English version analysis, after applying the exclusion criteria, we used the responses for 216 questionnaires but excluded those of four with outfit > 2.5. As in Rasch theory, an extreme score (0 or 60 for the CISS) on a questionnaire corresponds to an infinite ability measure (ability measure = symptoms level when using the CISS), which is impractical and also misleading in most situations.^24^ Winsteps excluded from the analysis four more completed questionnaires with scores of 0. Finally, the results of 208 questionnaires (38.0% female; mean age, 15.86 ± 1.61 years) were used in the Andrich's RSM analysis; this sample size is larger than the minimum recommended for DIF assessment. The mean CISS_VE_ score was 16.10 ± 9.50, and the range was 1 to 49. Table 7 compares the main psychometric properties of the two CISS versions.

**Table 7.** Comparison Between the Spanish and English Versions

	CISS_VE_	CISS
Parameter	(n = 429)	(n = 208)
Number of items	15	15
Response categories ordering	Ordered	Ordered
Person separation index (reliability)	2.33 (0.85)	2.31 (0.84)
PCA		
Raw variance explained by measure, %	45.8	46.2
Eigenvalue of the first contrast, logits	2.18	2.13
Number of items with infit outside 0.5 to 1.5	0	0
Number of items with outfit outside 0.5 to 1.5	0	1
Targeting	−1.37	−1.16

PCA, principal component analysis of the residuals; DIF, differential item functioning.

#### The DIF Contrast Was Below 0.50 for Every Item When Comparing CISS and CISS_VE_

Because the Kolmogorov–Smirnov test indicated a non-normal distribution of the CISS_VE_ scores, we ran a Kruskal–Wallis test followed by Dunn's multiple comparisons on the 429 completed questionnaires. This was designed to examine differences in CISS_VE_ performance according to gender and age: males from 9 to 17 years (boys), females from 9 to 17 years (girls), males from 18 to 30 years (young men), and females from 18 to 30 years (young women). The Kruskal–Wallis *H* test detected a significant among between the CISS_VE_ groups examined (*H*, 46.14; *P* < 0.001). Descriptive statistics are provided in Supplementary Table S2 and the significant differences detected by the Dunn's test are shown in Supplementary Table S3.

### Convergent Validity, Divergent Validity, and Repeatability

**Figure 4.**
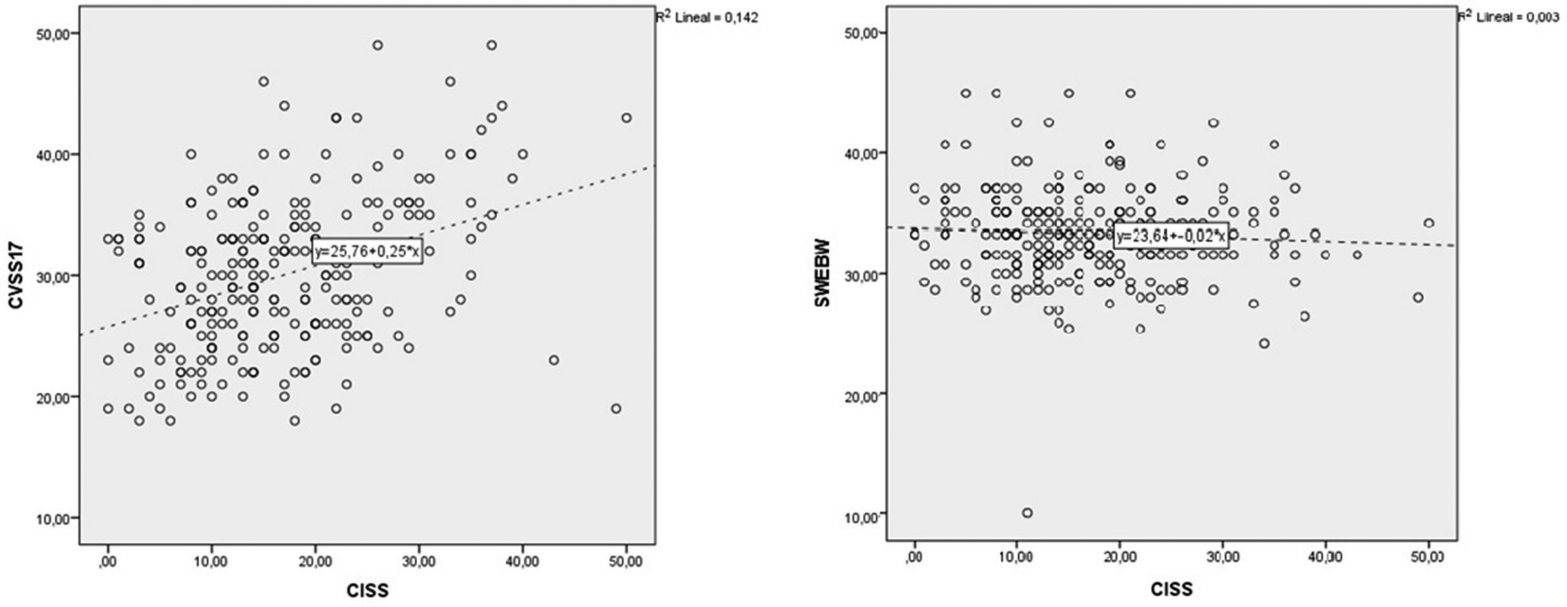
Scatterplots of correlations between the CISS_VE_ and the CVSS and between the CISS_VE_ and the shortened version of WEMWBS. The regression line is shown as a dotted line.

**Figure 5.**
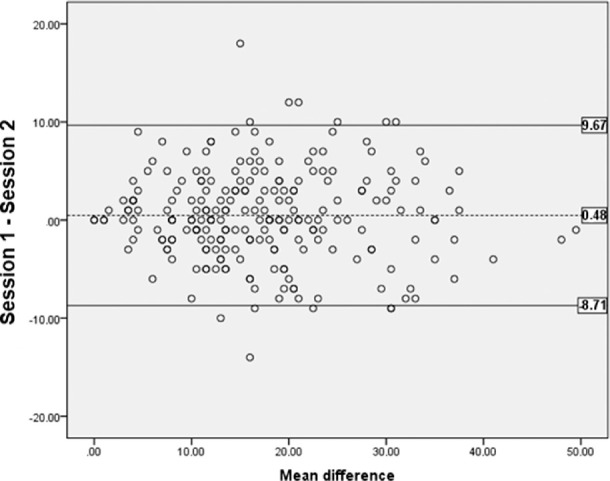
Bland–Altman plot for the CISS_VE_. The dotted line indicates the mean difference (MD) between scores obtained when completing the questionnaire on two occasions. The solid lines indicate the lower and the upper 95% limits of agreement (MD ± 1.96).

We calculated the Spearman rho correlation index between the CISS_VE_ and the CVSS at 0.34 (*P* < 0.001). No significant association was detected between the CISS_VE_ and the seven items covered in the shortened version of WEMWBS (Fig. 4), which indicates evidence of divergent validity. Correlation between the CISS_VE_ and the CVSS was weak (0.34)^32^ yet may be considered proof of convergent validity. For the subjects who completed the CISS_VE_ twice (test–retest time interval: 10.23 ± 3.40 days), the two-way single-measure ICC for test–retest repeatability was 0.878 (95% confidence interval, 0.845–0.905), and the CoR was 9.22. Figure 5 provides the Bland–Altman plot for the CISS_VE_. The mean difference between sessions was 0.48, and the limits of agreement including 95% of the differences were 9.67 and –8.71, so the CoR was 9.22. For four subjects, the difference in CISS score between sessions was over 10, which was considered by Rouse et al.^2^ as “significant and outside the range of normal variability.” By considering these four subjects as outliers and excluding them from the analysis, the CoR would improve to 8.51.

## Discussion

In this study, we present the Spanish version of the CISS, which shows psychometric properties similar to those for the English version. Our Rasch analysis also confirmed that the overall performance of the instrument is acceptable. A good-quality translation is an essential part of cross-cultural adaptation, but this does not mean that the translated version retains the psychometric properties of the original tool. Other authors propose a three-step process in which translation is followed by formal assessment of psychometric properties and validity and reliability testing.^8^^,^^11^

As recommended by Bradley and Massof,^33^ we directly compared item psychometric properties between the CISS_VE_ and the CISS to determine whether both tests worked in a similar way. According to their almost identical reliability and residual PCA results (Table 7), the psychometric performance of the CISS_VE_ proved similar to that of the CISS. A small difference was noted in targeting (–1.37 CISS_VE_ vs. –1.16 CISS), as the mean symptoms score in the English sample was one point higher than in the Spanish subjects. When comparing both versions, just one item (item 12: Do you feel a “pulling” feeling around your eyes when reading or doing close work?) showed an outfit value (1.86) far from Rasch model expectation (1.50). This was attributed to six respondents who scored 0 on every item except item 12, which they awarded a score of 1. Exclusion of these six subjects yielded an outfit value of 0.90. This indicates that the poor performance of item 12 may be attributed to the composition of our English sample. To confirm the equivalence between both versions, our DIF analysis confirmed that the CISS items had been optimally translated into Spanish (European).

We also compared the psychometric properties of the CISS_VE_ arising from Rasch analysis through Rasch model expectation^34^ (Table 6). Measurement precision was high, and more than six levels of convergence insufficiency symptoms could be distinguished in the study population. Further, although the CIRS group did not use Rasch analysis to develop the original questionnaire, just one item of our Spanish version (item 1: Do your eyes feel tired when reading or doing close work?) showed Rasch infit and outfit values under the minimum suggested by Khadka et al.^14^ (0.7). This indicates that this item's responses are too predictable despite being within the interval of 0.5 to 1.5, considered productive for measurement.^35^ Because the presence of one or two items with infit or outfit between 0.5 and 0.7 is deemed acceptable,^14^^,^^16^ item 1 was retained in the questionnaire without any modification.

Our results revealed that the CISS_VE_ is an instrument without DIF for gender and that minimal DIF^13^ according to age exists for only one item (item 14: Do you lose your place while reading or doing close work?). According to Khadka et al.,^14^ a notable DIF is >1.0 logits, so we could directly compare CISS_VE_ scores across these subgroups. Our analysis revealed higher CISS_VE_ scores in the young adults than in children (Supplementary Table S3). These differences may be due to a greater cognitive load associated with near vision in the young adults group exacerbating the symptoms normally induced by visual stressors.^36^

We also examined convergent validity by comparing the CISS_VE_ and the CVSS17. As predicted, a significant association emerged between them with a coefficient of correlation higher than 0.3 (Fig. 4). This value is the minimum recommended by Khadka et al.^14^ when assessing convergence validity. Further, it is the minimum suggested by the COSMIN guideline for systematic reviews of patient-reported outcome measures^37^ when evaluating construct validity by studying correlations with instruments measuring related but dissimilar constructs (like we did here). In addition, there was no correlation between the CISS_VE_ and the Spanish version of the WEMWBS,^29^ so our study provides some evidence of CISS_VE_ divergent validity, as it works in the expected manner.

Our CISS_VE_ showed a mean score (15.10 ± 10.13) that was comparable to those reported in studies conducted in similar populations, such as the adolescents assessed by Horan et al.^6^ (16.3 ± 11.4) and the university students used to develop the Portuguese version (CISS_VP_; 15.56 ± 8.86).^5^ These two studies provided mean scores for the entire group of participants instead of separating scores according to subjects’ visual problems. Furthermore, the mean score obtained in our sample (from a general population) is higher than those reported by Rouse et al.^2^^,^^38^ in adults and children with normal binocular vision recruited from a clinical population (11.3 ± 8.1 for adults and 10.4 ± 8.1 for children). As expected, our sample's mean score was lower than the values obtained in children^2^ and adults^38^ with symptomatic CI (37.3 ± 9.3 for adults and 29.8 ± 8.1 for children).

A main strength of our study was that we used Rasch analysis to analyze the psychometric properties of both the CISS_VE_ and the CISS and used data from the DIF analysis to compare the items of the two versions. However, our study also has the limitation of suboptimal targeting due to the use of the general population instead of a purposive sample.

Good targeting determines higher person reliability, so tests with poor targeting are worse at distinguishing between high and low performers. Thus, this could be a limitation of the CISS_VE_ because the targeting value (–1.37 logits) was lower than recommended (<–1.0) by Khadka et al.^14^ and Pesudovs et al.^16^ This suboptimal targeting is a typical issue of scales designed to measure symptoms^26^ when administered to the general population, as we did, and could indicate less measurement precision in subjects scoring far from the items’ distribution mean (i.e., subjects with fewer symptoms). However, the number of levels of performance (5.8) determined by the Wright method, which is a sample-independent technique derived from Rasch analysis,^39^ suggests the high reliability of the CISS_VE_. Given this sample-independent reliability along with the fact that clinicians and/or researchers usually focus on persons with scores closer to the items’ mean, we consider this CISS_VE_ mistargeting acceptable for its purpose.

Rasch analysis of the CISS_VE_ and the CISS suggested multidimensionality, as the variance explained by its measures was under 50% and the eigenvalue of the first strength was above 2.0. When we examined the six items included in the putative dimension arising from the PCA analysis (Table 3), we noted that they were those items exploring complaints other than visual and ocular symptoms. Accordingly, subjects provided answers about reading consciousness or reading performance differently than they did about symptoms. However, because this secondary dimension is closely related to the Rasch dimension, as shown by their disattenuated correlation coefficient (0.84), we can consider that the two dimensions are two different categories of the same trait (e.g., calculus and trigonometry items on a math test), so we may consider the CISS_VE_ a unidimensional tool for statistical purposes.^24^

In addition, to compare 95% limits of agreement, we selected the study by Rouse et al.,^2^ as it has a mean test–retest interval similar to ours (10.50 ± 7.50 days vs. 10.23 ± 3.40 days). As expected, limits of agreement were similar in our study (9.67 and –8.71) and in the study by Rouse et al.^2^ (9.0 and –7.6). According to Khadka et al.^14^ and Pesudovs et al.,^16^ the ICC reported in our sample (0.878) indicates high test–retest reliability. The CoR was 9.22 (8.51 without the four outliers), showing that, in the test–retest data, the probability of detecting a test–retest change in CISS score greater than 9.22 in the test population is 2.5%. These results imply that a clinician can be sure that a treatment has a significant impact on a patient's symptoms when finding a change of 10 points, the same value provided by Rouse et al.^2^ for the original CISS. Test–retest reliability is optimal when the limits of agreement are lower than the minimal clinically important difference (MCID) values, although lower values of a similar magnitude are considered positive.^16^ To sum up, the CoR of the CISS_VE_ would be good if we consider valid the value given for the CISS (10 points), but further studies are needed to define precise MCID values for this questionnaire.

Rasch analysis could be used to reengineer the CISS to enhance areas in which it here showed lower performance such as dimensionality or targeting. There are several options for this, such as collapsing some categories and/or deleting the second dimension found in the residual PCA. As several options are available, the clinical relevance of any change should be considered to assess the effectiveness of these improvements. For example, we could consider deleting the second dimension if the instrument becomes more sensitive to clinically meaningful changes.

## Conclusions

We developed a Spanish version of the CISS showing performance similar to that of the original version in English. We also identified some psychometric properties of the CISS that should be addressed in future studies to improve this instrument as a measure of near-vision-related symptoms.

## Supplementary Material

Supplement 1

Supplement 2

Supplement 3

## References

[1] Borsting EJ, Rouse MW, Mitchell GL, et al. Validity and reliability of the revised convergence insufficiency symptom survey in children aged 9 to 18 years. *Optom Vis Sci*. 2003; 80: 832–838. 14688547 10.1097/00006324-200312000-00014

[2] Rouse MW, Borsting EJ, Mitchell GL, et al. Validity and reliability of the revised convergence insufficiency symptom survey in adults. *Ophthalmic Physiol Opt*. 2004; 24: 384–390. 15315652 10.1111/j.1475-1313.2004.00202.x

[3] Borsting E, Rouse MW, De Land PN Prospective comparison of convergence insufficiency and normal binocular children on CIRS symptom surveys. Convergence Insufficiency and Reading Study (CIRS) group. *Optom Vis Sci*. 1999; 76: 221–228. 10333184 10.1097/00006324-199904000-00025

[4] Borsting E, Rouse MW, Deland PN, et al. Association of symptoms and convergence and accommodative insufficiency in school-age children. *Optometry*. 2003; 74: 25–34. 12539890

[5] Tavares C, Nunes AMMF, Nunes AJS, Pato MV, Monteiro PML Translation and validation of Convergence Insufficiency Symptom Survey (CISS) to Portuguese - psychometric results. *Arq Bras Oftalmol*. 2014; 77: 21–24. 25076368 10.5935/0004-2749.20140007

[6] Horan LA, Ticho BH, Khammar AJ, Allen MS, Shah BA Is the convergence insufficiency symptom survey specific for convergence insufficiency? A prospective, randomized study. *Am Orthopt J*. 2015; 65: 99–103. 26564934 10.3368/aoj.65.1.99

[7] Beaton DE, Bombardier C, Guillemin F, Ferraz MB Guidelines for the process of cross-cultural adaptation of self-report measures. *Spine J*. 2000; 25: 3186–3191. 10.1097/00007632-200012150-00014 11124735

[8] Gandek B, Ware JE Jr. Methods for validating and norming translations of health status questionnaires: the IQOLA Project approach. International Quality of Life Assessment. *J Clin Epidemiol*. 1998; 51: 953–959. 9817112 10.1016/s0895-4356(98)00086-9

[9] Gjersing L, Caplehorn JR, Clausen T Cross-cultural adaptation of research instruments: language, setting, time and statistical considerations. *BMC Med Res Methodol*. 2010; 10: 13. 20144247 10.1186/1471-2288-10-13 PMC2831007

[10] Muñiz J, Elosua P, Hambleton RK Directrices para la traducción y adaptación de los tests: segunda edición. *Psicothema*. 2013; 25: 151–157. 23628527 10.7334/psicothema2013.24

[11] Ware JE Jr, Gandek B. Methods for testing data quality, scaling assumptions, and reliability: the IQOLA Project approach. International Quality of Life Assessment. *J Clin Epidemiol*. 1998; 51: 945–952. 9817111 10.1016/s0895-4356(98)00085-7

[12] Adnan TH, Mohamed Apandi M, Kamaruddin H, et al. Catquest-9SF questionnaire: validation of Malay and Chinese-language versions using Rasch analysis. *Health Qual Life Outcomes*. 2018; 16: 5. 29304817 10.1186/s12955-017-0833-3 PMC5755437

[13] Khadka J, Huang J, Mollazadegan K, et al. Translation, cultural adaptation, and Rasch analysis of the visual function (VF-14) questionnaire. *Invest Ophthalmol Vis Sci*. 2014; 55: 4413–4420. 24917139 10.1167/iovs.14-14017

[14] Khadka J, McAlinden C, Pesudovs K Quality assessment of ophthalmic questionnaires: review and recommendation. *Optom Vis Sci*. 2013; 90: 720–744. 23873034 10.1097/OPX.0000000000000001

[15] McAlinden C, Pesudovs K, Moore J The development of an instrument to measure quality of vision: the Quality of Vision (QoV) questionnaire. *Invest Ophthalmol Vis Sci*. 2010; 51: 5537–5545. 20505205 10.1167/iovs.10-5341

[16] Pesudovs K, Burr J, Harley C, Elliott D The development, assessment, and selection of questionnaires. *Optom Vis Sci*. 2007; 84: 663–674. 17700331 10.1097/OPX.0b013e318141fe75

[17] Pesudovs K, Garamendi E, Elliott D The Quality of Life Impact of Refractive Correction (QIRC) Questionnaire: development and validation. *Optom Vis Sci*. 2004; 81: 769–777. 15557851 10.1097/00006324-200410000-00009

[18] WHO. Management of substance abuse: process of translation and adaptation of instruments. Available at: https://www.who.int/substance_abuse/research_tools/translation/en/. Accessed February 17, 2020.

[19] Wild D, Grove A, Martin M, et al. Principles of good practice for the translation and cultural adaptation process for patient-reported outcome (PRO) measures: report of the ISPOR task force for translating adaptation. *Value Health*. 2005; 8: 94–104. 15804318 10.1111/j.1524-4733.2005.04054.x

[20] Wu M, Adams R *Applying the Rasch Model to Psycho-Social Measurement: A Practical Approach*. Melbourne, Australia: Educational Measurement Solutions; 2007: 87.

[21] Wright BD Model selection: rating scale model (RSM) or partial credit model (PCM)?. *Rasch Meas Trans*. 1998; 12: 641–642.

[22] Khadka J, Gothwal VK, McAlinden C, Lamoureux EL, Pesudovs K The importance of rating scales in measuring patient-reported outcomes. *Health Qual Life Outcomes*. 2012; 10: 80. 22794788 10.1186/1477-7525-10-80 PMC3503574

[23] Linacre JM, Wright B. Winsteps help for Rasch analysis. Available at: URL: http://www winsteps com/index htm. Accessed June 27, 2019.

[24] Linacre J. *Winsteps Rasch Measurement Computer Program User's Guide*. Beaverton, OR: Winsteps.com; 2014.

[25] Marais I, Andrich D. Formalizing dimension and response violations of local independence in the unidimensional Rasch model. *J Appl Meas*. 2008; 9: 200–215. 18753691

[26] González-Pérez M, Susi R, Antona B, Barrio A, González E The Computer-Vision Symptom Scale (CVSS17): development and initial validation. *Invest Ophthalmol Vis Sci*. 2014; 55: 4504–4511. 24938516 10.1167/iovs.13-13818

[27] Petersen MA, Groenvold M, Bjorner JB, et al. Use of differential item functioning analysis to assess the equivalence of translations of a questionnaire. *Qual Life Res*. 2003; 12: 373–385. 12797710 10.1023/a:1023488915557

[28] Watt T, Barbesino G, Bjorner JB, et al. Cross-cultural validity of the thyroid-specific quality-of-life patient-reported outcome measure, ThyPRO. *Qual Life Res*. 2015; 24: 769–780. 25194574 10.1007/s11136-014-0798-1

[29] Castellvi P, Forero CG, Codony M, et al. The Spanish version of the Warwick-Edinburgh Mental Well-Being Scale (WEMWBS) is valid for use in the general population. *Qual Life Res*. 2014; 23: 857–868. 24005886 10.1007/s11136-013-0513-7

[30] Bland J, Altman D. Statistical methods for assessing agreement between two methods of clinical measurement. *Lancet*. 1986; 1: 307–310. 2868172

[31] Gonzalez-Perez M, Susi R, Barrio A, Antona B Five levels of performance and two subscales identified in the computer-vision symptom scale (CVSS17) by Rasch, factor, and discriminant analysis. *PLoS One*. 2018; 13: e0202173. 30153272 10.1371/journal.pone.0202173 PMC6112632

[32] Schober P, Boer C, Schwarte LA Correlation coefficients: appropriate use and interpretation. *Anesth Analg*. 2018; 126: 1763–1768. 29481436 10.1213/ANE.0000000000002864

[33] Bradley C, Massof RW. Validating translations of rating scale questionnaires using Rasch analysis. *Ophthalmic Epidemiol*. 2017; 24: 1–2. 28085560 10.1080/09286586.2016.1246667

[34] Khadka J, Gao R, Chen H, et al. Re-engineering the Hong Kong Quality of Life Questionnaire to assess cataract surgery outcomes. *J Refract Surg*. 2018; 34: 413–418. 29889295 10.3928/1081597X-20180326-01

[35] Linacre J What do infit and outfit, mean-square and standardized mean? *Rasch Meas Trans*. 2002; 16: 878.

[36] Gowrisankaran S, Nahar NK, Hayes JR, Sheedy JE Asthenopia and blink rate under visual and cognitive loads. *Optom Vis Sci*. 2012; 89: 97–104. 22051780 10.1097/OPX.0b013e318236dd88

[37] Prinsen CAC, Mokkink LB, Bouter LM, et al. COSMIN guideline for systematic reviews of patient-reported outcome measures. *Qual Life Res*. 2018; 27: 1147–1157. 29435801 10.1007/s11136-018-1798-3 PMC5891568

[38] Rouse M, Borsting E, Mitchell GL, et al. Validity of the Convergence Insufficiency Symptom Survey: a confirmatory study. *Optom Vision Sci*. 2009; 86: 357–363. 10.1097/OPX.0b013e3181989252 PMC2779473 19289977

[39] Wright B Separation, reliability and skewed distributions: statistically different levels of performance. *Rasch Meas Trans*. 2001; 14: 786.

