# Supplementary material for: Spanish Cross-Cultural Adaptation and Rasch Analysis of the Convergence Insufficiency Symptom Survey (CISS)
Source: Transl Vis Sci Technol. 2020 Mar 23;9(4):23. doi: 10.1167/tvst.9.4.23 (PMC7396165; doi:10.1167/tvst.9.4.23)
Supplement: Supplement 1 [file tvst-9-4-23_s001.pdf]

Traducción inversa consensuada	Back translation AB	Back translation PAUL SAPSFORD	Back traslation CONSENSUADA
**Escala sobre síntomas de Insuficiencia de Convergencia**	**Convergence insufficiency symptom scale**	**Scale of symptoms of insufficient convergence**	**Convergence insufficiency symptom scale**
DT Item	DT Item	DT Item	DT Item
**Instrucciones:** Las siguientes preguntas se refieren a cómo te sientes mientras lees o trabajas de cerca	**Instructions.** Please respond to the following questions about how you feel when you are reading or doing other close work	**Instructions for the clinic:** Read the following instructions and then each question exactly how it is written. If the patient responds yes, please qualify the answer with how frequently. Don't put examples.<br>**Instructions for the patient:** The following questions refer to how you feel while you read or work close-up.	**Instructions.** Please respond to the following questions about how you feel when you are reading or doing other close work
1. ¿Notas tus ojos cansados?	1. Do your eyes feel tired?	1. Do you feel your eyes are tired?	1. Do your eyes feel tired?
2. ¿Notas incomodidad en tus ojos?	2. Do you have discomfort?	2. Do you feel discomfort in your eyes?	2. Do you have discomfort in your eyes?
3. ¿Te duele la cabeza?	3. Do you get headaches?	3. Does your get headaches?	3. Do you get headaches?
4. ¿Te entra sueño?	4. Do you get tired?	4. Do you feel tired?	4. Do you feel tired?
5. ¿Pierdes la concentración?	5. Do you have difficulty concentrating?	5. Do you lose concentration?	5. Do you lose your concentration?
6. ¿Te cuesta recordar lo que has leído?	6. Do you find it hard to remember what you've read?	6. Do you find it difficult to remember what you have read?	6. Do you find it difficult to remember what you have read?
7. ¿Ves doble?	7. Do you see double?	7. Do you see double? (double vision)	7. Do you see double?
8. ¿Te parece que las palabras se mueven, se mezclan o flotan sobre el texto?	8. Does it feel like the words move, mix together or float above the text?	8. Do you think the words move, they mix or float over the text?	8. Does it feel like the words move, merge or float on the page?
9. ¿Te parece que lees lento?	9. Do you feel like you read slowly?	9. Do you think you read slowly?	9. Do you feel like you read slowly?
10. ¿Te duelen los ojos?	10. Do your eyes hurt?	10. Do your eyes hurt?	10. Do your eyes hurt?
11. ¿Se te irritan los ojos?	11. Do your eyes itch?	11. Do your eyes get irritated?	11. Do your eyes feel sore?
12. ¿Tienes sensación de "tirantez" alrededor de los ojos?	12. Do you feel tightness around the eyes?	12. Do you have the sensation of tightness around the eyes?	12. Do you feel tightness around the eyes?
13. ¿Notas que las palabras se ponen borrosas o que se enfocan y desenfocan?	13. Do the words blur or come in and out of focus?	13. Do you notice the words go blurry or focus and defocus	13. Do you notice the words blur or come in and out of focus?
14. ¿Te pierdes de línea al leer?	14. Do you lose your line?	14. Do you lose the line you are reading?	14. Do you lose the line you are reading?
15. ¿Tienes que releer la misma línea de texto?	15. Do you have to re-read the same line?	15. Do you re-read the same line of text?	15. Do you have to re-read the same line?
**CATEGORIAS DE RESPUESTA**	**REPLY CATEGORIES**	**REPLY CATEGORIES**	**REPLY CATEGORIES**
1. Nunca	1. Never	1. Never	1. Never
2. Muy pocas veces	2. Hardly ever	2. Rarely	2. Rarely
3. Algunas veces	3. Sometimes	3. Sometimes	3. Sometimes
4. Muchas veces	4. Often	4. Frecuently	4. Often
5. Siempre	5. Always	5. Always	5. Always

Supplementary Doc S1. Written summary of the expert committee review. Green background indicates that we chose the option proposed by that translator and orange background shows that the traslation was achieved by consensus after
